# 1428. Clinical Characteristics and Management of Nontuberculous Mycobacterial Infections in the Finger Lakes Region of New York

**DOI:** 10.1093/ofid/ofac492.1257

**Published:** 2022-12-15

**Authors:** Michael Croix, Ghinwa Dumyati, Alexandra Adams, Dwight Hardy, Paul Levy, Emil Lesho, Sonal Munsiff

**Affiliations:** University of Rochester, Rochester, New York; University of Rochester Medical Center Division of Infectious Diseases, Rochester, New York; University of Rochester Medical Center, Canandaigua, New York; University of Rochester Medical Center, Canandaigua, New York; University of Rochester Medical Center, Canandaigua, New York; Rochester General Hospital, Rochester, New York; Univ. of Rochester, Rochester, New York

## Abstract

**Background:**

Nontuberculous Mycobacteria (NTM) are ubiquitous in the environment and associated with pulmonary and extra-pulmonary infections. Evaluation and treatment of these infections is complex and prolonged, involving oral, intravenous and inhaled therapies, although not all patients with NTM need treatment. We reviewed the clinical characteristics and referral patterns of NTM patients in our institutions to evaluate resource needs.

**Methods:**

We retrospectively identified all patients who had NTM isolated from 2 referral labs servicing the Finger Lakes Region of NYS, from April 1, 2018 thru March 31, 2020. Demographic, comorbidities, culture, and treatment data were collected from medical records. We compared comorbidities, clinical symptoms, and species among patients with pulmonary and extra-pulmonary NTM. Pearson’s chi-squared and student t-test were performed where appropriate for statistical analysis.

**Results:**

155 NTMs were isolated from 149 patients, 128 from pulmonary and 27 from extra-pulmonary sites. Patients with pulmonary NTM were more likely to be older, female, and have underlying lung disease (Table 1). Patients with extra-pulmonary NTM were more likely to have immunodeficiency. *Mycobacterium avium* complex (MAC) was isolated more frequently in pulmonary samples and rapidly growing mycobacteria more frequently from extra-pulmonary samples (Table 2). Disease site specific symptoms were more common than constitutional symptoms in both groups. 36% of patients with pulmonary NTM were treated compared with 70% of patients with extra-pulmonary NTM (Table 3). 69% were referred to Pulmonologists, and 43% to infectious diseases for evaluation and management.

**Table 1:**
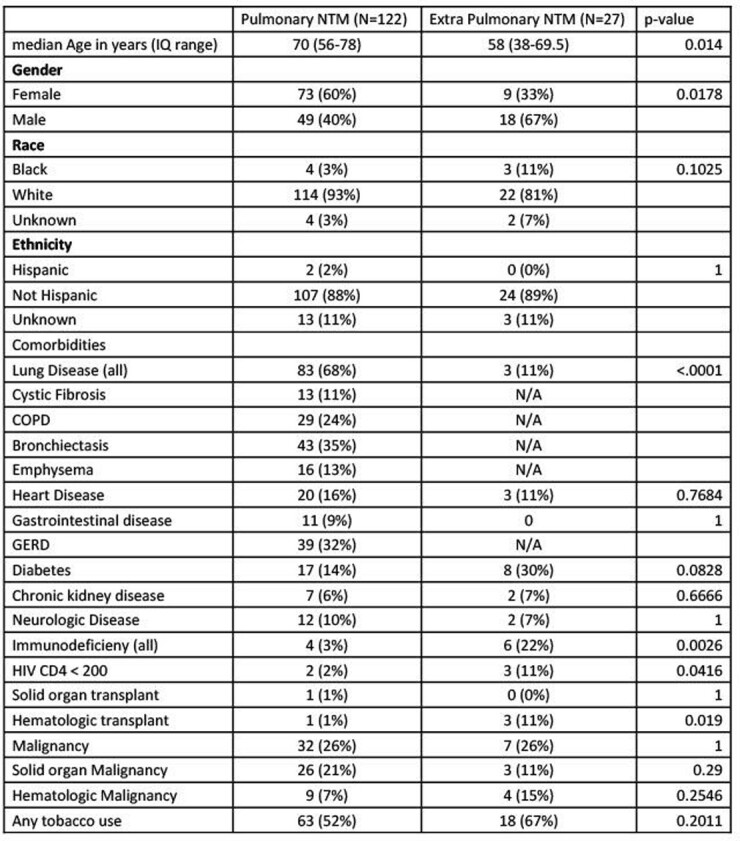
Demographics

Demographic data for patients with pulmonary and extrapulmonary NTM

**Table 2:**
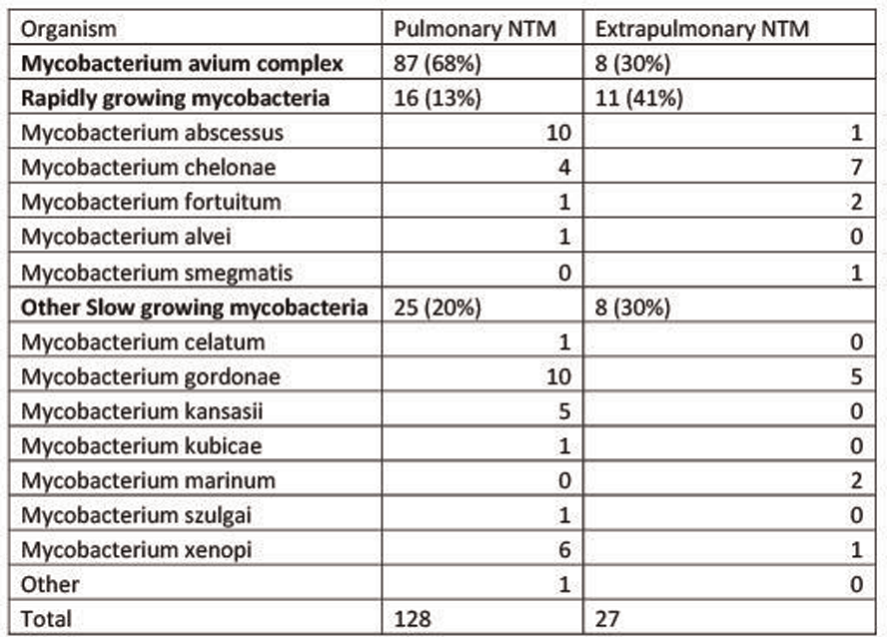
Mycobacterium species

Mycobacterium species isolated from patients with pulmonary and extrapulmonary NTM

**Table 3:**
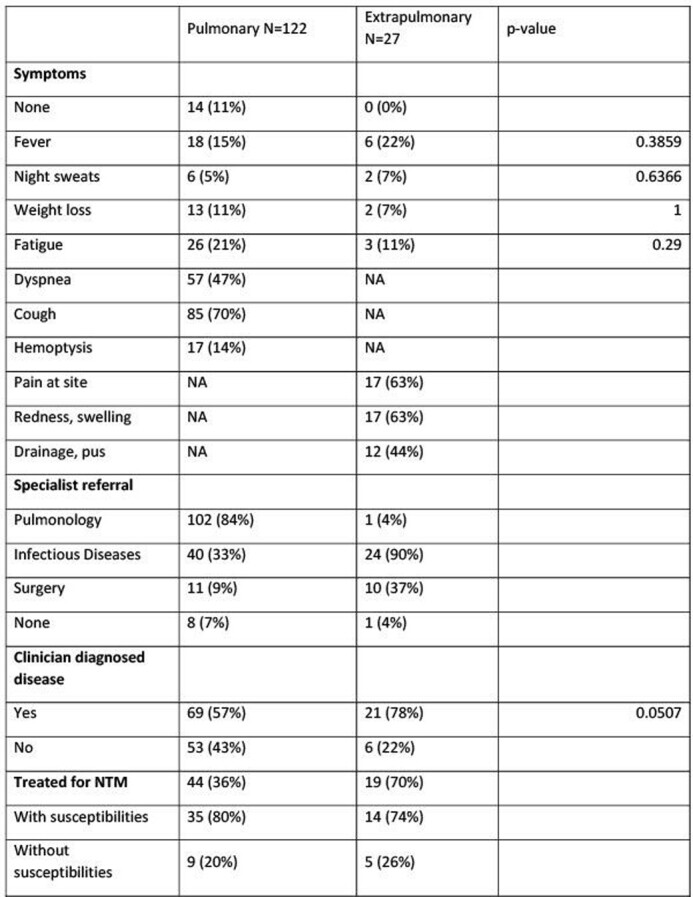
Clinical characteristics

Clinical characteristics of patients with pulmonary and extrapulmonary NTM including symptoms, referral to specialists, whether patients were diagnosed with disease by a clinician, and how many were treated.

**Conclusion:**

Though not all patients with NTM isolated from culture require treatment, most of the patients in our cohort had extensive evaluation, those on treatment required multidisciplinary care, and many not on treatment require ongoing monitoring. Knowing the volume and clinical characteristics of NTM patients in our region has helped to identify the needed resources for developing a comprehensive regional NTM center of Excellence. Other institutions can similarly assess their volume to plan for the increasing incidence of these infections.

**Disclosures:**

**Ghinwa Dumyati, MD**, Pfizer: Grant/Research Support.

